# Quantitative Insights into the Genetic Mechanisms of Crop Heterosis

**DOI:** 10.1002/ggn2.202500030

**Published:** 2025-11-29

**Authors:** Zhiwu Dan, Yunping Chen, Wenchao Huang

**Affiliations:** ^1^ State Key Laboratory of Hybrid Rice Key Laboratory for Research and Utilization of Heterosis in Indica Rice the Ministry of Agriculture The Yangtze River Valley Hybrid Rice Collaboration & Innovation Center College of Life Sciences Wuhan University Wuhan China

**Keywords:** compound traits, genomics‐downstream variations, heterosis, hybrid crops, inheritance patterns, prediction

## Abstract

Heterosis, the universal phenomenon in which F_1_ hybrids exhibit superior performance compared to their parental lines, is widely exploited in modern agriculture for improving crop yield, yet its genetic mechanisms remain incompletely understood. The contributions of parental genomic sequence variants to heterosis have been extensively investigated. Recent advances in transcriptomics, proteomics, and metabolomics offer new quantitative perspectives on the molecular basis of crop heterosis. This review summarizes current evidence on inheritance patterns: additive, partially dominant, dominant, and overdominant effects. We highlight that the contributions of these effects to heterosis vary by genotypes, traits, tissues, populations, developmental stages, omics datasets, growth environments, and species. The additive effect, which can be utilized to predict heterosis for F_1_ hybrids and accelerate the breeding of hybrid crops, has been identified as a predominant inheritance pattern in complete diallel crosses. We propose that integrating multi‐omics data and quantitative analysis of inheritance patterns can deepen our understanding of its genetic mechanisms and accelerate the breeding of elite hybrid varieties. This approach provides a framework for predicting breeding and the rational design of high‐yield crops.

## Graphical abstract

Inheritance patterns of genomics‐downstream molecules, including transcripts, proteins, and metabolites, are analyzed in hybrid crops. The additive effect, which describes mid‐parent level, is a predominant inheritance pattern in crop heterosis and can be used to improve breeding efficiency of high‐yield hybrid varieties.

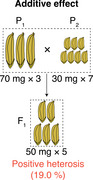

## 1 Introduction

Heterosis, synonymy of hybrid vigor [1, 2, 3, 4], refers to the phenomenon where F_1_ hybrids outperform their genetically divergent parents in morphological, physiological, and chemical traits. These traits include biomass (e.g., yield, weight, stature, productiveness, height, area, size, and content), fertility, speed, resistance (to diseases, pathogens, or unfavorable conditions), quality, developmental timing, and coloration, which are measurable through observable or detectable parameters. The first systematic description of heterosis was provided by Charles Robert Darwin in plants 150 years ago [5]. The exploitation of heterosis in maize started in the United States before 1930, with hybrid varieties yielding nearly six times that of the inbred lines [6, 7]. The utilization of rice heterosis in China, which is through a hybrid rice breeding program initiated in 1964, results in hybrids with 20%–30% yield advantages over conventional varieties [8]. To facilitate the exploitation of heterosis in crop breeding, genetic models have been proposed to explain its mechanisms at the genomic level. Among these, the dominance (and pseudo‐overdominance) [9, 10, 11, 12, 13], overdominance [1, 14, 15, 16, 17], and epistasis [18, 19, 20] models emphasize the complementation of deleterious alleles, heterozygosity of loci, and inter‐locus interactions, respectively. Numerous reviews have summarized advances in uncovering the genetic underpinnings of heterosis, driven largely by progress in molecular biology technologies [6, 21, 22, 23, 24, 25, 26, 27, 28, 29, 30, 31, 32, 33, 34, 35]. However, no consensus has been reached, and the molecular mechanisms underlying crop heterosis remain elusive.

**FIGURE 1:**
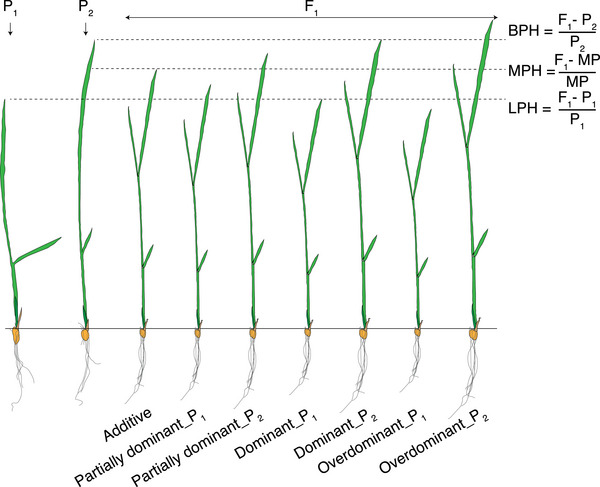
Four inheritance patterns in F_1_ hybrids. Based on phenotypic values of F_1_ hybrids relative to their two parents, inheritance patterns can be classified as additive, partially dominant, dominant, and overdominant effects. Using rice seedling length as an example, the additive effect describes that the F_1_ hybrid shows no significant difference from the mid‐parent value (the average of the two parents, P_1_ and P_2_) but differs significantly from both P_1_ and P_2_. The partially dominant effects describe that the seedling length of the F_1_ hybrid significantly deviates from the mid‐parent value and is intermediate between the mid‐parent value and P_1_ or P_2_. The dominant effects describe that the seedling length of the F_1_ hybrid resembles that of either P_1_ or P_2_ and significantly differs from the mid‐parent value. Finally, the overdominant effects describe that the F_1_ hybrid is significantly shorter than the low parent or longer than the high parent. Furthermore, the degrees of heterosis for seedling length can be assigned to low‐parent heterosis (LPH), mid‐parent heterosis (MPH), and better‐parent heterosis (BPH) relative to the values of P_1_, mid‐parent, and P_2_, respectively.

East stated that “*the problem of heterosis is the problem of the inheritance of quantitative characters*” [1]. These “quantitative characters” are quantitatively measurable phenotypes or molecular expression levels. They exhibit distinct inheritance patterns (Figure 1), including additive, partially dominant, dominant, and overdominant effects [20, 32]. It is important to interpret these patterns cautiously in the context of the dominance and overdominance models mentioned above. The additive effect describes a scenario where the phenotypic values or molecular levels of F_1_ hybrids are at mid‐parent values (MPVs) and are significantly different from either parent. Partially dominant effects mean that the levels of F_1_ hybrids deviate from the MPV toward one parent but do not reach the parental level. Depending on the direction of deviation, these are further categorized as partially dominant_female or partially dominant_male. Dominant effects indicate that the F_1_ hybrids are statistically indistinguishable from one parent (either female or male). Overdominant effects represent that the F_1_ hybrids exceed the high‐parent value or fall below the low‐parent value, designated as overdominant_female or overdominant_male. Additionally, cases with insignificant differences between parents or between F_1_ hybrids and parents/MPVs are classified as unclassifiable effects [36]. Furthermore, the degrees of heterosis are commonly assessed for phenotypic data by calculating low‐parent heterosis (LPH), mid‐parent heterosis (MPH), and better‐parent heterosis (BPH).

**FIGURE 2:**
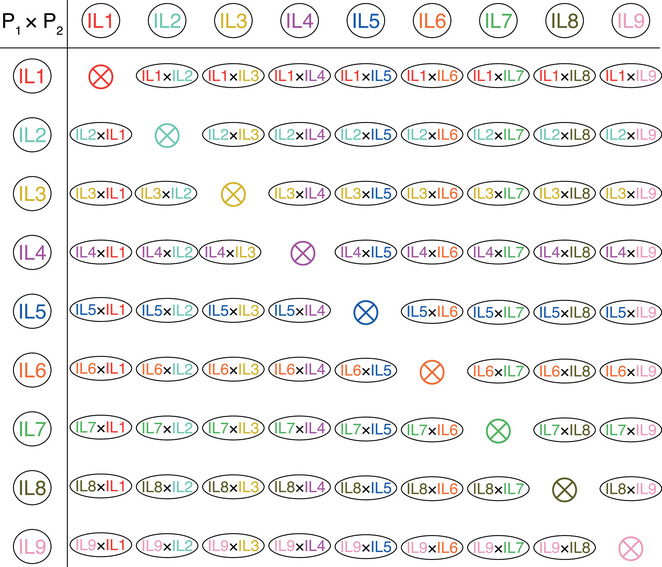
A complete diallel cross design. As shown as an example, nine representative inbred lines (ILs) were selected as parental lines. Each IL was crossed with the remaining eight ILs, including reciprocal hybrids. With nine parents, the expected number of F_1_ hybrids is 72. A subset of this population, excluding reciprocal hybrids, constitutes a half diallel cross design.

Heterosis is the fruit of hybridization [37], the design of which is critical for accurately assessing inheritance patterns in F_1_ hybrids. Dozens of heterosis studies report on fewer than ten F_1_ hybrids derived from several parental lines [38, 39, 40, 41, 42, 43, 44, 45, 46, 47, 48, 49, 50]. Crossing designs for larger hybrid populations include complete or half diallel cross (Figure 2), backcross or testcross, random mating, intergroup or factorial mating, and three‐ or four‐way cross. The complete diallel cross design, involving all possible pairwise crosses, was reported in tomato over 50 years ago using 18 parents (18 × 17 F_1_ hybrids) [51]. Later, two sunflower populations were constructed using complete diallel designs (15 × 14 and 8 × 7 F_1_ hybrids) [52], and a similar approach was applied in rice using 18 representative inbred lines [53, 54, 55]. The half diallel cross design, which omits reciprocal crosses, has been widely adopted in crops such as tomato (10 × 9/2 F_1_ hybrids)^22^, wheat (13 × 12/2 F_1_ hybrids) [56], rice (11 × 10/2, 10 × 9/2, and 9 × 8/2 F_1_ hybrids) [57, 58], and maize (12 × 11/2 F_1_ hybrids) [59]. Backcross F_1_ (BC_1_F_1_) populations, generated by crossing recombinant inbred lines (RILs) to their parental lines or other inbred lines, have been developed in maize [60] and rice [61, 62, 63, 64]. In tomato, introgression lines carrying single chromosome segments from wild species were crossed with the cultivar M82 to compare trait values between homozygous and heterozygous plants [65]. Similarly, testcross populations, using a limited number of testers (e.g., Zhong413 and IR64 in rice, B73 in maize) crossed with RILs or inbred lines, have been constructed in rice [66] and maize [67, 68]. Random hybridization among high‐generation RILs (F_9_) has been designed to generate F_1_ hybrids in rice [69, 70, 71]. Similarly, in maize, RILs derived from a single F_1_ hybrid were randomly divided into two groups, and F_1_ hybrids were randomly produced through inter‐group crosses to evaluate ten agronomic traits [72]. In maize, inbred lines have been categorized into dent and flint groups, with F_1_ hybrids produced through inter‐group crossing [73, 74, 75, 76, 77, 78]. In rice, male sterile lines have been crossed with potential restorer varieties to investigate heterosis of important agronomic traits [79, 80, 81, 82]. Factorial mating designs, both complete and incomplete, have been employed in various crops, including wheat (120 females × 15 males) [83, 84, 85], sunflower (15 females × 8 males) [52], and maize (1428 females × 30 males) [86]. Unlike these designs, the three‐way F_1_ hybrids, generated by crossing a single cross tester (F_1_, X × Y, a cytoplasmic male sterile line) with randomly selected F_3_ progenies (male parents), have been developed in rye [87]. The four‐way crosses, namely the double‐cross F_1_ hybrid ((A × B) × (C × D)) with four parental lines, have been reported in maize [7]. Furthermore, to investigate heterosis in historically utilized hybrid crops across breeding stages, elite F_1_ hybrid cultivars derived from different inbred lines have been pooled for analysis in rice [88, 89] and maize [90].

Among these hybridization designs, the complete diallel cross design requires all parents to have normal fertility, making it less efficient than using male sterile lines in hybridization and more challenging than other designs. However, it offers advantages for the comprehensive, quantitative analyses of heterosis. If *N* parental lines are used for hybridization, the number of F_1_ hybrids is N*(*N*‐1), with each parent contributing to 2*(*N*‐1) F_1_ hybrids. This design ensures equal representation of each parental line in the hybrid population, eliminating bias due to an unequal number of hybrids. Importantly, the balanced population structure also allows for accurate estimation of contributions from both female and male parents to the F_1_ hybrids, a feature that distinguishes it from other designs. The contributions of additive, partially dominant (female or male), dominant (female or male), and overdominant (female or male) effects can be assessed, and the predominant inheritance patterns can be determined by comparing their associations with heterosis.

As heterosis is a property of quantitative traits or characters [91], its interpretation may benefit from quantitative strategies that go beyond genomic sequence variation [29]. Here, we summarize findings from genomics‐downstream analyses to provide quantitative insights into the genetic mechanisms of crop heterosis. Although environmental factors [92, 93, 94], including the microbiome [95, 96, 97], influence crop heterosis, this review focuses on genetic determinants.

## 2 Dissecting Heterosis: Component and Compound Traits

Heterosis can be calculated for either a component trait or a compound trait in F_1_ hybrids. Heterosis of compound traits is the product of their component traits at the phenotypic level [18, 51, 91, 98, 99], which may result from epistatic interactions between genomic loci. Compound traits consist of two or more component traits. As previously described [19], plant height is determined by internode number and internode length [18, 100]; grain yield is determined by grain number per panicle, panicles per plant, grain weight, and seed setting rate [57, 101]; leaf area is determined by leaf length and leaf width; grain weight is determined by grain length, grain diameter, grain thickness, and grain density [102]; and growth rate is determined by cell division rate and cell enlargement rate.

Strong heterosis for compound traits in F_1_ hybrids can occur even when parents exhibit similar phenotypic values for the compound traits, provided that their component traits differ reciprocally [19]. This phenomenon has been attributed to reciprocal inequality of gene function between parents [91], a concept termed “mock‐dominance” [18, 19]. For heterosis to arise, the two parents must differ in their component traits, with neither parent exhibiting high values for all components [51, 98]. Additive effects of component traits, including additive‐by‐additive interactions, consistently contribute to positive heterosis in compound traits [13, 36, 51, 91].

Component traits are often negatively correlated, and the expression of each component is constrained to avoid reduced fitness in F_1_ hybrids [51, 91, 103]. In other words, heterosis for compound traits in F_1_ hybrids is optimized when parental differences in the expression of component traits remain within a restricted range [54, 104], before incompatibility barriers emerge. Extreme heterosis of component traits can lead to disharmony, such as dwarfism or sterility. Expanding on the gene balance hypothesis [105, 106, 107], maintaining an appropriate balance among component traits or their associated molecules is crucial for heterosis. Therefore, identifying and analyzing component traits is essential for a deeper understanding of crop heterosis [51, 103].

## 3 Inheritance Patterns shaping Crop Heterosis

### 3.1 Inheritance Patterns of Agronomic Traits

At the phenotypic level, grain or fruit yield in certain crops is determined by the product of grain/fruit number and weight. In tomato, multiplicative interactions between various genetic effects of fruit number and size/weight, including additive‐by‐partially dominant [20, 91, 103], additive‐by‐dominant [20, 51], additive‐by‐overdominant [99], partially dominant‐by‐partially dominant [20, 91, 103], partially dominant‐by‐dominant [91], and partially dominant‐by‐overdominant effects [20], have been reported to contribute to yield heterosis (Table 1). In bean, the additive effect was more influential than the dominant effect for traits such as seed number per plant, seed weight, and pod number per plant, while additive‐by‐dominant interactions accounted for a major role in yield heterosis [98]. Similarly, in rice, the additive effect of component traits, which behaved in a multiplicative interaction manner, was proposed as the foundation of heterosis for compound traits [55].

**TABLE 1:** Inheritance patterns of agronomic traits, expressed genes, proteins, and metabolites in maize, rice, and tomato.

Species	Inheritance patterns of agronomic traits	Inheritance patterns of expressed genes	Inheritance patterns of proteins	Inheritance patterns of metabolites
Maize	Additive and overdominant effects [112].	Additive [114, 115, 116, 117, 118, 120, 121, 122, 123, 124, 125, 126], partially dominant [119], dominant [116, 117, 118, 126], and overdominant [116, 117, 118, 119, 126] effects.	Additive [52, 139, 141] and overdominant [140] effects.	Additive, dominant, and overdominant effects [144].
Rice	Additive [111], partially dominant [69, 109, 111], dominant [69, 88, 110, 111], overdominant [69, 108], additive‐by‐additive [69], additive‐by‐dominant [69], dominant‐by‐dominant [69, 108], and additive‐by‐overdominant [63] effects.	Additive [36, 127, 129, 130, 132, 134, 135], partially dominant [131, 133], dominant [128, 133], and overdominant [128, 133] effects.	Dominant and overdominant effects [133].	Additive effect [36, 145].
Tomato	Dominant [65], overdominant [65], additive‐by‐partially dominant [20, 91, 103], additive‐by‐dominant [20, 51], additive‐by‐overdominant [99], partially dominant‐by‐partially dominant [20, 91, 103], partially dominant‐by‐dominant [91], and partially dominant‐by‐overdominant [20] effects.	—	—	Additive and dominant effects [142].

With the utilization of DNA markers in heterosis studies, associations between quantitative trait loci (QTLs) and trait values have been elucidated. In tomato, the overdominant effect was prevalent for reproductive traits, including fruit yield, while the dominant effect was observed across all investigated traits [65]. In rice, partially dominant, dominant, and overdominant effects at the single‐locus level, along with additive‐by‐additive, additive‐by‐dominant, and dominant‐by‐dominant digenic interactions contributed to heterosis in the elite hybrid “Shanyou63” [69]. In the analysis of two backcross F_1_ populations from 9311 (*indica*) and DT713 (*japonica*), additive‐by‐overdominant effects were more important than partially dominant and dominant effects [63]. Overdominant and pseudo‐overdominant effects were identified as key contributors to heterosis for yield, grain number per panicle, and grain weight in rice, whereas dominant‐by‐dominant interaction was important to heterosis for tiller number per plant and grain weight [108]. A study of 1495 widely cultivated hybrid rice varieties concluded that the dominant effect significantly contributed to heterosis of 38 agronomic traits [88]. Additionally, genotyping and phenotyping of 10074 F_2_ rice lines indicated that a partially dominant effect influenced heterosis for yield‐related traits [109]. For heterosis of grain quality‐related traits, which differ from traits like yield and plant height, most QTLs showed a dominant effect and were distinct from mid‐parent values in rice [110]. The heterosis gene *OsMADS1*, which simultaneously regulates grain yield and quality, showed additive and partially dominant effects for grain length and grain weight but a dominant effect for the percentage of chalky grain in the heterozygous state [111], suggesting distinct inheritance patterns for different traits controlled by the same heterosis gene. In maize, overdominant effects were detected for most QTLs associated with grain yield, while an additive effect was observed for grain moisture in F_1_ hybrids [112]. For wheat, contributions to grain yield heterosis were partitioned as follows: additive‐by‐additive (50%), additive‐by‐dominant (21%), dominant‐by‐dominant (13%), and dominant effects (15%) [113]. Taken together, the contributions of these inheritance patterns to crop heterosis vary across studies. Deeper investigation of heterosis from the perspective of heterosis‐related QTLs or genes, integrating well‐designed population structures, fully‐recorded phenotypic data, and comprehensive genomic variants, remains needed.

### 3.2 Inheritance Patterns of Expressed Genes

Transcriptomic analyses of F_1_ hybrids and their parental lines have elucidated inheritance patterns of expressed genes in maize and rice, providing valuable clues to the genetic mechanism of crop heterosis.

In maize, transcriptomic profiling of immature ear tissues revealed a positive correlation between the proportion of additively expressed genes and yield heterosis [114]. Another study found that all possible inheritance patterns were observed for gene expression levels in F_1_ hybrids from B73 and Mo17, with 78% of detected genes exhibiting an additive effect [115]. Similarly, additive, dominant, and overdominant effects were observed in hybrid embryos and endosperms from UH005 (flint) and UH301 (dent) six days after fertilization [116, 117, 118]. For plant height heterosis in six maize F_1_ hybrids, over 50% of genes showed overdominant effect, while 26%, 12.6%, and 10.2% exhibited partially dominant, dominant, and additive effects, respectively [119]. In contrast, transcriptomic analysis of primary roots in B73, Mo17, and their reciprocal F_1_ hybrids revealed additive expression in about 90% of genes [120]. Maize endosperm genes also displayed predominantly additive expression across three developmental stages [121]. The majority (∼70%) of differentially expressed genes (DEGs) in seedling tissues of six F_1_ hybrids also followed an additive effect [122]. For grain yield heterosis in maize, the additive effect was predominant among heterosis‐associated genes localized in pericentromeric regions [123]. Large‐scale transcriptomic profiling (approximately 40 000 genes across 23 tissues) in B73, Mo17, and their F_1_ hybrid further confirmed that additive effects were more common than non‐additive effects, particularly for genes with large expression variation or single‐gene expression patterns [124, 125]. Analysis of 395 allele‐specific expression genes in reciprocal F_1_ hybrids from B73 and Mo17 indicated that the additive effect was the majority (30.45%–50.32%), followed by dominant (13.25%–34.08%) and overdominant effects (4.59%–19.70%) [126]. In short, while no uniform conclusion emerges, the additive effect at the transcriptomic level appears to play a crucial role in maize heterosis.

In rice, small RNA expression analysis in young seedlings showed that 17% (140 in total) and 18% (157 in total) of small RNAs exhibited an additive effect in reciprocal F_1_ hybrids of 93‐11 and Nipponbare [127]. Among 16 gibberellin‐related genes analyzed in rice seedlings, 13 displayed dominant or overdominant effects [128]. Differential expression analysis of seedling samples from PA64S, 93‐11, and their F_1_ hybrid revealed additive expression in 66% of the 3488 DEGs. In a comparison of Nipponbare, 93‐11, and their F_1_ hybrid, 44% and 56% of the 2416 DEGs displayed additive and nonadditive effects, respectively [129]. Small RNA profiling in flag leaves and panicles (at the grain‐filling stage) of super hybrid rice “Liangyoupeijiu” indicated that 80.56% (286 of 355) had expression levels consistent with mid‐parental values [130]. Genome assembly of parental lines R900 and Y58S (of the heterotic hybrid Y900) identified partially dominant effects as the primary genetic basis of heterosis in Y900 [131]. Comparative transcriptomics analysis between parents (XH220 and PS2) and F_1_ hybrids was performed on young panicles of diploid and autotetraploid rice [132]. The results indicated that 38.2% and 46.2% of DEGs were classified as additive in diploid and tetraploid hybrids, respectively [132]. In transcriptomics analysis of heterosis for salt tolerance in the super hybrid rice CY1000 (from Guangxiang24S and R900), the authors focused on non‐additive genes and found that the percentages for overdominant, dominant, and partially dominant genes under salinity stress conditions were 33%, 24%, and 43%, respectively [133]. Transcriptomic results of four F_1_ hybrids with strong yield heterosis showed that the additive effect of *OsDCL2*, *Pi33*, and *Pi5*, and the dominant effect of *Pid2* played crucial roles in rice heterosis [134]. Among the allele‐specific expression genes identified in these hybrids, 46.8%, 43.4%, and 31.3% of genes showed an additive effect in young seedlings, young panicles, and filling panicles, respectively [134]. The combination of RNA‐sequencing and ribosome profiling provided a closer look at gene inheritance patterns at transcriptional and translational levels [135]. The results confirmed that the additive effect was predominant at both the transcriptional (16453 genes, 93.3%) and translational (12815 genes, 96.8%) levels. Furthermore, most single‐parent expressed genes (89.3% of the transcriptome and 94.1% of the translatome) behaved additively in the hybrid of Z04A (*japonica* female parent) and ZHF1015 (*indica* male parent) [135]. Thus, the additive effect also stands out among other inheritance patterns in rice heterosis.

For the different inheritance patterns of expressed genes in F_1_ hybrids, epigenetic variations in parents, including DNA methylation levels [134, 136], chromatin accessibility [137], and histone modifications [136], are involved in the regulation of gene expression and crop heterosis. Beyond genomic variants, these epigenomic modifications play important roles in determining gene expression levels in F_1_ hybrids relative to their parents. A comprehensive investigation of the associations between genomic variants, epigenetic modifications, and inheritance patterns of expressed genes is essential to elucidate the mechanisms of crop heterosis.

### 3.3 Inheritance Patterns of Proteins

Protein inheritance patterns have been extensively studied in maize. In a proteomic analysis of 3.5‐day‐old primary roots from the F_1_ hybrid of UH301 (dent) and UH002 (flint), 49% (150 of 304) of detected proteins exhibited nonadditive effects, differing from the average levels of their parental inbred lines [138]. In contrast, among 597 proteins detected in embryos of F_1_ hybrids from UH005 (flint) and UH250 (dent), 456 exhibited an additive effect [139]. Similarly, 91% of the 970 identified proteins displayed an additive effect in the seminal roots of reciprocal F_1_ hybrids from B73 and Mo17 [52]. In embryos of germinated seeds from five maize F_1_ hybrids, 46.30%–80.06% of detected proteins showed nonadditive effects, with overdominant effects (59.62%) predominating [140]. Principal component analysis of maize seeds further supported additive regulation, with five heterotic F_1_ hybrids clustering between their parents in score plots, suggesting that additively balanced networks underpin maize heterosis [141]. In proteomic analysis of roots from hybrid rice CY1000, among the non‐additive proteins, 42% and 58% exhibited overdominant and dominant effects upon salinity stress, respectively [133]. Compared to transcriptomic analyses of crop heterosis, proteomic analyses are still nascent, and more investigation is needed to draw firm conclusions at the proteomic level.

### 3.4 Inheritance Patterns of Metabolites

With the rapid development of gas‐chromatograph or liquid‐chromatograph mass spectrometry profiling technologies, inheritance patterns of metabolites have also been explored. In tomato, QTL analysis of primary metabolic traits revealed that most metabolic QTLs (254 of 332) exhibited a dominant effect, while a smaller proportion (61 of 332) showed an additive effect, and an overdominant effect was negligible [142]. Theoretical metabolic network analyses proposed that antagonistic additive‐by‐additive interaction could contribute to heterosis [143]. Maize root metabolome comparisons identified additive, dominant, and overdominant effects, with no single pattern dominating [144]. In rice, untargeted metabolomics of seedling‐stage reciprocal F_1_ hybrids revealed hybrid metabolic profiles closely aligned with parental means, consistent with additive inheritance [145]. Similarly, both genes and metabolites exhibited additive effects across developmental stages in rice [36]. In *Brassica juncea*, the additive effect was more prevalent in buds and siliques (52.77%–97.14%) than in leaves (47.37%–80%) for primary and secondary metabolites [146]. In summary, consistent with findings at phenotypic, transcriptomic, and proteomic levels, the additive effect at the metabolomic level appears important in crop heterosis.

### 3.5 A Quantitative Model for Heterosis of Compound Traits

**FIGURE 3:**
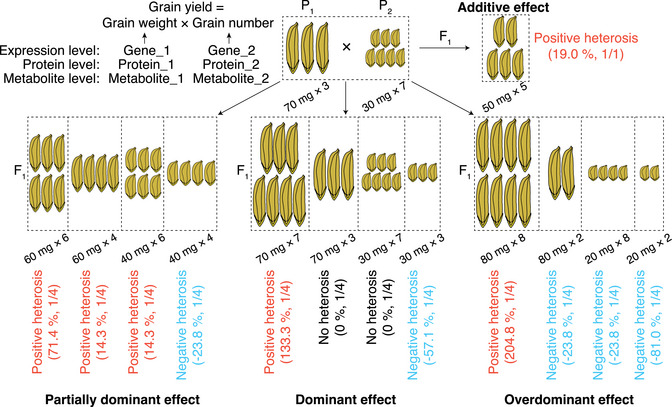
Model of different inheritance patterns of two‐component traits and their contributions to heterosis for grain yield. Using rice as an example, this model illustrates how different inheritance patterns of grain weight and grain number contribute to heterosis for grain yield. For clarity, grain yield is simplified to two component traits, and parental lines (P_1_ and P_2_) are assumed to have equal grain yield (hypothetical value). Grain weight and grain number values are manually assigned based on inheritance patterns. Positive heterosis (heterosis > 0) and negative heterosis (heterosis < 0) are defined. The degrees of heterosis for grain yield are calculated through the equation: heterosis (%) = 100 × (F_1_–P_1_)/P_1_. For partially dominant, dominant, and overdominant effects, three possible degrees of heterosis arise, corresponding to four combinations of grain weight and grain number. Since values of grain number and grain weight are directly or indirectly associated with transcriptomic, proteomic, or metabolomic changes, the model can be adapted to inheritance patterns of heterosis‐associated genes, proteins, or metabolites.

Integrating phenotypic, transcriptomic, proteomic, and metabolomic findings, we propose a model linking different inheritance patterns to heterosis of compound traits (Figure 3). For simplicity, we assume rice grain yield comprises only two component traits—grain weight and grain number—and that the two parental ILs have equal grain yield. We assign hypothetical values, which may not exist in actual rice varieties, to each IL's grain weight and grain number. The model delineates four inheritance patterns and their corresponding effects on heterosis for grain yield. When parental grain yields are equal, positive heterosis in the F_1_ hybrid is invariably observed due to additive‐by‐additive effects between the two component traits. With partially dominant, dominant, or overdominant effects, three possible degrees of heterosis for grain yield in F_1_ hybrids may arise. For partially dominant effects, the percentage of positive heterosis for grain yield is 75%, with the degrees of heterosis at 71.4% and 14.3%. The percentage of negative heterosis for grain yield is 25%, with a degree of 23.8%. For dominant effects, the percentage of positive heterosis changes to 25%, with a degree of 133.3%. There may be half F_1_ hybrids that have no heterosis for grain yield. The percentage of negative heterosis is 25% and the degree is 57.1%. When considering overdominant effects, the percentage of positive heterosis for grain yield remains at 25%, while 75% of F_1_ hybrids show negative heterosis, at degrees of 23.8% and 81.0%.

With partially dominant, dominant, and overdominant effects, the proposed model shows that relatively high heterosis values for grain yield occur in 25% of potential F_1_ hybrids, while the degree of negative heterosis can be as severe as 81.0% under overdominant effects. We speculate that these extreme values occur at low probabilities in nature, whereas intermediate values are likely more frequent. We assume the same inheritance patterns for the two component traits to show the simplest combinations. Different inheritance patterns of these component traits can be combined to produce varying degrees of heterosis. Furthermore, differences in parental grain yield can alter heterosis outcomes in F_1_ hybrids, particularly when multiple inheritance patterns are involved.

Because trait values are directly or indirectly influenced by changes in transcriptomic, proteomic, or metabolomic levels, this model can be extended to inheritance patterns of heterosis‐associated genes, proteins, or metabolites. Using gene expression as an example, we assume that gene_1 and gene_2 are associated with grain weight and grain number, respectively. In P_1_ and P_2_, there exist reciprocally unequal expressions of gene_1 and gene_2, corresponding to reciprocal inequality in grain number and grain weight. The expression levels of gene_1 and gene_2 show different inheritance patterns in F_1_ hybrids. Consequently, the values of grain number and grain weight change correspondingly, and the degrees of yield heterosis vary across the different genetic effects.

## 4 Predicting Crop Heterosis with Multi‐Omics Inheritance Patterns

Genomics‐downstream molecules, including transcripts, proteins, and metabolites, serve as intermediates linking genomic information to agronomic traits in crops [32]. Compared to genomic variants, these molecules exhibit stronger associations with phenotypic variation. Gene expression levels and metabolite concentrations frequently correlate with phenotypes of hybrid crops [142]. In maize, predictive accuracies for seven biomass‐ and bioenergy‐related traits in testcross F_1_ hybrids were comparable between 130 leaf metabolites (0.60–0.80) and 56110 SNPs (0.72–0.81) [68]. Similarly, metabolites from young maize roots successfully predicted the fresh weight of F_1_ hybrids in distinct field trials [147]. While metabolites show high predictive ability, transcriptomic data outperformed both genomic and metabolomic data in predicting maize heterotic traits such as dry matter yield [148] and grain yield [149] in other studies. In wheat, SNP markers and flag leaf metabolites were used to predict hybrid performance. Metabolomic data (predictive accuracy: 0.15–0.74) showed lower predictive accuracy than genomics (0.32–0.89), and the addition of metabolomics to genomics did not improve accuracy [85]. Conversely, in rice, metabolite profiles from parental seedling tissues robustly predicted hybrid phenotypes, heterosis, and specific combining ability across different populations [53], growth environments [145, 150], and developmental stages [151]. Additionally, the predictability of yield was nearly doubled when metabolomic data were applied in rice hybrids, compared to that of genomic data alone [152].

The prediction of hybrid performance has been partially explored under different inheritance patterns. In maize, incorporating additive and dominant effects into metabolite levels of 120 root metabolites improved biomass prediction in F_1_ hybrids [153]. In rice, metabolomic additive effect predicted grain weight and yield heterosis [145, 150, 154], and transcriptomic additive effect also contributed to heterosis prediction [155]. Furthermore, additive and partially dominant effects of expressed genes or metabolites have been applied to predict heterosis of agronomic traits in both maize and rice [36]. These findings suggest that the additive effect of genomics‐downstream molecules strongly correlates with heterosis in F_1_ hybrids and exhibits high predictive ability [36, 145]. Although most studies use arithmetic means to represent the additive effect, geometric means may better reflect molecular levels of F_1_ hybrids and warrant further investigation [1, 11].

## 5 Conclusions and Future Perspectives

Elucidating the molecular mechanisms underlying crop heterosis remains a key challenge in plant genetics. Although diverse inheritance patterns have been proposed to explain heterosis, conclusions often differ due to variations in genotypes, traits, tissues, developmental stages, growth environments, species, omics data types and completeness, and analysis thresholds or strategies. Investigating inheritance patterns for genomics‐downstream molecules based on complete diallel crosses contributes to resolving these discrepancies. Although additive and other genetic effects have been identified and partially explain heterosis, accurate prediction requires the development of robust predictive models. Achieving this will depend on the comprehensive integration of representative parental lines, well‐designed crosses, detailed dissection of compound traits, multi‐tissue and multi‐omics data, time‐series analyses, localized environmental factors, and appropriate statistical approaches. Collectively, quantitative analyses of genomics‐downstream molecules, together with environmental and epigenetic insights, will deepen our understanding of crop heterosis and accelerate the breeding of elite hybrid varieties, contributing to global food security.

## Author Contributions

Z.D. conceived the review. Z.D. and Y.C. prepared the figures. Z.D., Y.C., and W.H. wrote the manuscript.

## Funding

The National Natural Science Foundation of China (31801439, 32472185, and 32101667), the China Postdoctoral Science Foundation (2022T150500 and 2023T160497), the Key Research and Development Program of Hubei Province (2022BFE003), and the Hubei Agriculture Science and Technology Innovation Center Program.

## Conflicts of Interest

The authors declare no conflicts of interest.

## Peer Review

The peer review history for this article is available in the Supporting Information for this article.

## Supporting information




**Supplementary Information**: Record of Transparent Peer Review

## Data Availability

The authors have nothing to report.
