# Supplementary material for: Quantitative Insights into the Genetic Mechanisms of Crop Heterosis
Source: Adv Genet (Hoboken). 2025 Nov 29;6(4):e00030. doi: 10.1002/ggn2.202500030 (PMC12747548; doi:10.1002/ggn2.202500030)
Supplement: Supplementary file 1 — Supplementary Information: Record of Transparent Peer Review [file GGN2-6-e00030-s001.docx]

Record of Transparent Peer Review

ggn2.202500030

Quantitative Insights into the Genetic Mechanisms of Crop Heterosis

Data submitted: 16-May-2025

1st Editorial Decision: 25-July-2025

1^st^ Revision Received: 29-Aug-2025

2^nd^ Editorial Decision: 19-Nov-2025

Last Revision Received: 20-Nov-2025

Accepted: 20-Nov-2025

Revision with responses: 29-Aug-2025

COMMENTS TO AUTHOR:

Reviewer #1: The present review manuscript focuses on the genetic mechanisms underlying heterosis, integrating multi-omics data (transcriptomic, proteomic, and metabolomic) that build upon genomic insights. This approach offers both theoretical and practical value to the field. The following questions and suggestions are proposed:

Response: We sincerely thank you for the positive assessment and for providing these constructive suggestions, which have greatly helped us improve our manuscript.

1. While it is essential to provide background knowledge, the fundamental design of heterosis research and the genetic models influencing heterosis—corresponding to parts 2, 3, Figures 1 and 2 in the manuscript—are not directly relevant to the focus of this article, which reviews the genetic mechanisms of heterosis from a multi-omics perspective. It is evident that this information is well-established within the field. I believe that a more effective strategy would be to ensure brevity by incorporating this content into the introduction section rather than presenting it as a formal research report.

Response: We thank the reviewer for this excellent suggestion. In the revised manuscript, we have moved Figure 1 and Figure 2 into the Introduction section to provide a more concise background. We retained these figures because the distinction between genetic models of heterosis and inheritance patterns (e.g., dominance model vs. dominant effect) can often be misinterpreted, and Figure 1 helps to clarify these concepts. Furthermore, since the accurate assessment of the contributions of different inheritance patterns to heterosis is fundamentally dependent on hybridization designs, we believe Figure 2 effectively underscores the importance of the complete diallel cross.

2. Although epistasis is mentioned in the introduction section, its impact on the genetic mechanisms of heterosis remains inadequately addressed. The author may consider expanding upon this discussion, particularly regarding epistasis's role within multi-omics datasets.

Response: We appreciate the reviewer for pointing this out. In our review, we primarily use the term "epistasis" to describe inter-locus interactions at the genomic level. When discussing genomics-downstream molecules (transcripts, proteins, metabolites) and agronomic traits, we refer to "interactions" among these molecules or component traits, which can be analogously treated as manifestations of epistasis (Lines 144, 157, 171, 178, 180, 190, 195, and 307).

3. The manuscript lacks clear conclusions concerning its primary research objectives. In the conclusion section, while acknowledging that multiple factors influence heterosis analysis—which must be considered for accurate phenotypic predictions—the authors' statements remain overly generalized. A concise overview highlighting significant advancements in utilizing multi-omics data to elucidate heterosis should be provided for clarity and emphasis.

Response: We thank the reviewer for this valuable suggestion. To address this, we have inserted brief summaries to synthesize findings (Lines 122-134, 210-214, 240-242, 273-282, 298-300, and 315-317). We have also revised the Conclusions section to provide a more concise and impactful overview of the advancements in using multi-omics data for investigating heterosis, highlighting how these contributions lead to more accurate assessments of inheritance patterns and robust predictive models for improving the breeding efficiency of elite hybrid crops (Lines 408-417).

Reviewer #2:

Make abstract more scientific, covering all essential components.

Response: We thank you for this reminder. We have comprehensively strengthened the Abstract to enhance its clarity, scientific rigor, and completeness (Lines 9-26).

Keywords, must be other than the words given in the title of the article.

Response: As suggested, we have revised our keywords (Lines 27-28).

Author mentioned some genetic models but most recent models are not mentioned focusing subject matter.

Response: We appreciate your suggestion. Our review is specifically focused on the inheritance patterns of genomics-downstream molecules and agronomic traits to provide quantitative insights into crop heterosis. Therefore, while we acknowledge their importance, we have not included a comprehensive discussion of the most recent genomic models of heterosis.

There is need to incorporate more in-depth information on inheritance pattern.

Response: We have provided detailed information on different inheritance patterns in the Introduction section and have used Figure 1 to improve clarity on this fundamental concept (Lines 51-80).

Some more details need to incorporate on "Cross Design", to improve your stance.

Response: We have added new content to the manuscript to further emphasize the critical role of cross design for the accurate assessment of different inheritance patterns (Lines 122-134).

There is need to incorporate more details on heterosis of complex traits especially quality, and how these traits will be studied?

Response: This is an excellent point. We have now incorporated a discussion on the heterosis of quality traits. For instance, we highlight that complex traits like grain quality are often regulated by QTLs with dominant effects. We also cite the example of the heterosis gene *OsMADS1*, which controls both yield and quality, exhibiting distinct inheritance patterns (additive/partially dominant for grain length/weight vs. dominant for chalkiness) for different traits in its heterozygous state. This new content can be found in the revised manuscript (Lines 199-206).

Add some techniques employed during post-genomic time, to strengthen the subject matter.

Response: To address this, we have reorganized the third section of the manuscript into distinct subsections based on the analyzing technologies employed, which significantly improves the overall clarity and structure of our presentation.

Add some tabulated information in the revised manuscript.

Response: As suggested, we have included a new table (Table 1) that summarizes the inheritance patterns of agronomic traits, expressed genes, proteins, and metabolites in major crops like maize, rice, and tomato.

Cite some latest literature

Özkan, R., M. Bayhan, L. Yorulmaz, M. Öner, Ö. Albayrak, M. Yildirim and C. Akinci. 2024. Genotypic Responses of Some Cereal Species to Speed Breeding Conditions. Pak. J. Agri. Sci., Vol. 62(1), 9-18; 2025 ISSN (Print) 0552-9034, ISSN (Online) 2076-0906 DOI:10.21162/PAKJAS/25.45 <http://www.pakjas.com.pk>

Abbas, M.M., M.A. Ghani, K. Ziaf, T.N. Khan, M.M. Jahangir, F.S. Awan, S. Nawaz and M.M. Hussain. 2023. Development and characterization of F1 hybrid tomato by inter and intraspecific hybridization. Pak. J. Agri. Sci., Vol. 61(1), 199-207; 2024 ISSN (Print) 0552-9034, ISSN (Online) 2076-0906 DOI:10.21162/PAKJAS/24.218 <http://www.pakjas.com.pk>

Mushtaq, T., A.N. Shahzad, N. Hussain, S. Saleem, M.K. Qureshi and S.A.H. Bukhari. 2023. Growth, yield performance, and nitrogen use efficiency of wheat as affected by nitrogen fertilization under arid agro-climatic conditions. Pak. J. Agri. Sci., Vol. 60(3), 445-454; 2023 ISSN (Print) 0552-9034, ISSN (Online) 2076-0906 DOI:10.21162/PAKJAS/23.108 <http://www.pakjas.com.pk>

Response: We are grateful to the reviewer for suggesting these recent articles. We have incorporated some latest publications from 2022-2025, to expand the discussion in our revised manuscript.

Reviewer #4: Heterosis is widely exploited in modern agriculture for improving crop yield. However, its genetic mechanisms remain elusive. The authors reviewed from the inheritance patterns of phenotypes and molecular levels in F1 hybrids, cross designs of hybrid populations, heterosis of complex and component traits, contributions of different inheritance patterns to heterosis of complex traits to the prediction of heterosis using postgenomic molecules, and try to provide quantitative insights into the genetic mechanisms of crop heterosis. About this MS, I have following comments and suggestions:

Response: Thank you for your time and for providing these valuable suggestions to help us improve our work.

1, The major limitation of this manuscript is that, the authors predominantly adopts a textbook-style conceptual statement, and merely enumerate research findings without providing any analytical synthesis or original conclusion derived from these data.

Response: We thank the reviewer for this important suggestion. We have added brief summaries in the manuscript to synthesize the enumerated findings (Lines 122-134, 210-214, 240-242, 273-282, 298-300, and 315-317). Furthermore, we have revised the Conclusions section to highlight these quantitative insights, particularly their contributions to improving the breeding efficiency of elite hybrid crops.

2, The inheritance patterns of additive, partially dominant, dominant, and over-dominant effects are fundamental concepts in genetics that do not require graphical illustration. Figure 1 should be deleted as these are already well-established in standard genetic theory. 3, The complete diallel cross design is a conventional hybridization method in genetics that does not require graphical illustration. As it occupies considerable space without enhancing conceptual clarity, we recommend deletion of Figure 2.

Response: Thank you for the reminder. We have moved both figures into the Introduction section. We retained Figure 1 because it helps prevent the common misinterpretation between genetic models (e.g., dominance model/hypothesis) and inheritance patterns (e.g., dominant effect). We kept Figure 2 to visually emphasize our point that the accurate assessment of these inheritance patterns is critically dependent on robust hybridization designs like the complete diallel cross.

4, In Page11-12, A model linking inheritance patterns to heterosis of complex traits at the phenotypic level (Figure 3) is proposed, and it is mentioned that this model could also be extended to molecules at the transcriptomic, proteomic, and metabolomic levels. But how to do that, the authors haven't give any clue. In addition, the manuscript fails to provide a clear explanation of how heterosis is calculated within the proposed model.

Response: Thank you for identifying this lack of clarity. We have now amended the manuscript to provide a clear explanation on how to expand the proposed model to heterosis-associated molecules (e.g., genes, proteins, metabolites) (Lines 359-368). Regarding the calculation of heterosis, we have explicitly defined the formulas for low-parent heterosis (LPH), mid-parent heterosis (MPH), and better-parent heterosis (BPH) in Figure 1 (Lines 65-67) to ensure it is clearly presented to the readers.
